# Phenotypic Plasticity of *Staphylococcus aureus* in Liquid Medium Containing Vancomycin

**DOI:** 10.3389/fmicb.2019.00809

**Published:** 2019-04-16

**Authors:** Mengdi Rong, Xuyang Zheng, Meixia Ye, Jun Bai, Xiangming Xie, Yi Jin, Xiaoqing He

**Affiliations:** ^1^College of Biological Sciences and Biotechnology, Beijing Forestry University, Beijing, China; ^2^Center for Computational Biology, College of Biological Sciences and Technology, Beijing Forestry University, Beijing, China

**Keywords:** phenotypic plasticity, bivariate GWAS, *Staphylococcus aureus*, vancomycin, whole-genome sequencing

## Abstract

Phenotypic plasticity enables individuals to develop different phenotypes in a changing environment and promotes adaptive evolution. Genome-wide association study (GWAS) facilitates the study of the genetic basis of bacterial phenotypes, and provides a new opportunity for bacterial phenotypic plasticity research. To investigate the relationship between growth plasticity and genotype in bacteria, 41 *Staphylococcus aureus* strains, including 29 vancomycin-intermediate *S. aureus* (VISA) strains, were inoculated in the absence or presence of vancomycin for 48 h. Growth curves and maximum growth rates revealed that strains with the same minimum inhibitory concentration (MIC) showed different levels of plasticity in response to vancomycin. A bivariate GWAS was performed to map single-nucleotide polymorphisms (SNPs) associated with growth plasticity. In total, 227 SNPs were identified from 14 time points, while 15 high-frequency SNPs were mapped to different annotated genes. The *P*-values and growth variations between the two cultures suggest that non-coding region (SNP 738836), *ebh* (SNP 1394043), drug transporter (SNP 264897), and *pepV* (SNP 1775112) play important roles in the growth plasticity of *S. aureus*. Our study provides an alternative strategy for dissecting the adaptive growth of *S. aureus* in vancomycin and highlights the feasibility of bivariate GWAS in bacterial phenotypic plasticity research.

## Introduction

Phenotypic plasticity reflects the adaptation of organisms and provides the ability of a given individual to develop different traits in different environments. Phenotypic plasticity was first applied to plants and quickly penetrated into various disciplines (Corona et al., 2016; Savvides et al., 2017). How bacteria respond to environmental disturbances is complicated and cannot be predicted by traditional experiments. A given genotype cannot be well adapted to all environments, and the response of genetic variance to environmental pressure may be very low (Anurag, 2001). Phenotypic plasticity enables bacteria to counteract the threat of the environment to growth and propagation (Chevin and Hoffmann, 2017). Bacteria in a changeable environment show greater adaptation to a novel condition (Opijnen et al., 2016). For example, Antarctic lake strains have better phenotypic plasticity than ancestral polar sea strains at high salinities (Rengefors et al., 2015). Phenotypic plasticity is a way for a species to adapt to environmental changes and a main driving force of adaptive evolution (Gibbin et al., 2017).

*Staphylococcus aureus* is an important human pathogen (Laupland, 2013). The abuse of antibiotics has increased the drug resistance of *S. aureus* (Nikam et al., 2017). Vancomycin is the last line of defense for *S. aureus* infections. Vancomycin-resistant strains adapt to the antibiotic environment by thickened cell walls, reduced toxicity, and an altered rate of autolysis (Gardete and Tomasz, 2014). Studies have identified several genes and mutations that affect the evolution of vancomycin-resistant *S. aureus* (Shoji et al., 2011; Hu et al., 2016). The response of an individual species to natural selection or other competitions depends on phenotypic changes, which involve both genetic variation and phenotypic plasticity (Charmantier et al., 2008; Chevin et al., 2010). Phenotypic plasticity plays an important role in stabilizing community function in the face of environmental changes, and is essential for the survival of bacteria (Beier et al., 2015). However, the relationship between phenotypic plasticity and drug resistance in *S. aureus* is unknown.

Genome-wide association study (GWAS) provides a new opportunity to explore the response of genes or chromosomal regions to different phenotypes (Visscher et al., 2017). In the past decade, GWAS have been successfully applied to human disease analyses and genetic studies of different organisms (Saxena et al., 2007; Power et al., 2016). Due to the increasingly cheap and high-throughput sequencing technologies, bacterial GWAS provides a new and effective method for understanding the phenotypic genetic mechanism of bacteria (Timothy and Ruth, 2014; Power et al., 2016). A GWAS study of methicillin-resistant *S. aureus* (MRSA) has successfully indentified numbers of toxicity-related SNPs and highlights the potential of bacterial GWAS in clinical research (Laabei et al., 2014). Recently, SNP–SNP epistatic interactions analysis was performed to dissect fitness costs in the evolution of MRSA (Yokoyama et al., 2018), and a new mapping framework was developed to unravel the genetic mechanism of bacterial interactions (Jiang et al., 2018). Taking *S. aureus* as an example, we investigated the associations between growth plasticity and single-nucleotide polymorphisms (SNPs). The growth data of 41 *S. aureus* strains, including 29 vancomycin-intermediate *S. aureus* (VISA) strains, in the absence or presence of vancomycin were obtained. The significant SNPs related to the growth plasticity of different strains were analyzed in a bivariate GWAS. These results not only provide an alternative strategy for dissecting VISA development, they also highlight the feasibility of bivariate GWAS for the analysis of phenotypic plasticity in bacteria.

## Materials and Methods

### Bacterial Strains and Pre-cultivation

All *S. aureus* strains were obtained by 60-day *in vitro* vancomycin treatment in our previous study (Wang et al., 2016). Strains’ background is listed in Supplementary Table S1; 41 parental strains from 12 sequence types were treated with vancomycin for 60 days as described (Wang et al., 2016). Each parental strain (S1–S9 and S11–S42) was incubated on brain heart infusion (BHI) (Oxoid, Basingstoke, United Kingdom) agar plates with vancomycin (Sigma–Aldrich, St. Louis, MO, United States) of 50% initial minimum inhibitory concentration (MIC) at 37°. The strains were passaged to fresh medium with same vancomycin concentration every 24 h. After 4 days treatment, MICs were re-determined and the concentration of vancomycin was increased to 50% of new MIC. The *in vitro* vancomycin treatment was repeated every 4 days for 60 days. Finally, 41 strains (S1’–S9’ and S11’–S42’) with elevated MICs were obtained and preserved at −80°. Before growth plasticity experiment, single colonies were transferred to BHI culture medium and shaken overnight at 37°C.

### Growth Curve Assay and Maximum Growth Rate (MGR) Determination

Overnight cultures were inoculated into fresh BHI medium with (2 μg/ml) or without vancomycin (Sigma–Aldrich, St. Louis, MO, United States) at a concentration of 6 × 10^3^ CFUs/ml. Growth curve experiments were carried out in 96-well plates (Corning, New York, NY, United States) in 150-μl volumes in triplicate. Cell growth was monitored over 48 h by OD_600_ on a microplate reader (Infinite M200 PRO, Tecan, Switzerland). Fourteen data points were collected: 1, 2, 4, 6, 8, 10, 12, 16, 20, 24, 30, 36, 42, and 48 h. Data were plotted to a smooth curve. The MGR was determined as described previously (Daniele et al., 2016).

### Whole-Genome Sequencing

Genomic DNA was extracted using a TIANamp Bacteria DNA Kit (TIANGEN, Beijing, China) according to the manufacturer’s protocols. In total, 41 *S. aureus* genomes were genotyped using an Illumina HiSeq 4000 instrument (Illumina Inc., San Diego, CA, United States) at Allwegene (Beijing, China); *S. aureus* subsp. aureus NCTC 8325 (NC_007795.1) was used as the reference strain. Quality control of the raw sequencing data included the removal of low-quality and adapter reads, calculating the sequencing error rate, Q20 and Q30 statistics, and GC content analysis. The sequence data were then compared to *S. aureus* subsp. aureus NCTC 8325 genome using the BWA mapper v0.7.8 (Li, 2013); the initial alignment results were obtained in BAM format. The alignment results were then ordered using SAMtools software package v0.1.18 (Li et al., 2009) and repeat reads were marked. SAMtools was also used to detect SNPs and the obtained SNPs were filtered.

### Bivariate GWAS

FastStructure software v1.0 (Anil et al., 2014) was used to characterize the population structure. The whole analysis of population structure was reflected as an exhaustive exploration of *k*. Given the certain subpopulation number of *k*, *meanQ* was calculated for each strain and for each subpopulation in the mapping population, inferring the posterior probability of subpopulation with each strain was belonging to. The Akaike information criterion (AIC) value was calculated for each *k*. The *meanQ* with best *k* value (minimum AIC) thus offered results of population structure.

In this study, the multivariate linear mixed model (LMM) was used to calculate the *P-*value to determine interesting SNPs at each time point using GEMMA software v0.97 (Zhou and Stephens, 2014). Growth data from both untreated and vancomycin-treated group formed the bivariate phenotype, which differs hugely from the univariate phenotype. Therefore, LMM developed for univariate phenotype cannot be applied to analyze correlated phenotype directly; methods allowing LMM algorithm specific to multivariate and correlated phenotype [multivariate LMMs (mvLMMs)] are in urgent need. GEMMA software could extend a standard LMM to mvLMMs, which simultaneously analyze correlated phenotypes but without losing mapping accuracy.

Like traditional LMM model, population structure and kinship were all considered to be implemented in multivariate LMM. GEMMA fit a mvLMM in the following form:

𝐘=⁢𝐗⁢B+𝐒⁢A+𝐐𝐕+𝐔+𝐄;

$$𝐔∼M\,N_{n\times 2}\,\left(𝟎,𝐊,V_{g}\right),𝐄∼M\,N_{n\times 2}\,\left(𝟎,I_{n\times n},V_{e}\right).𝐔∼M\,N_{n\times 2}\left(𝟎,𝐊,V_{g}\right),𝐄∼M\,N_{n\times 2}\,\left(𝟎,I_{n\times n},V_{e}\right).$$

Consider the bivariate phenotype from twogroups, *n* strains in mapping population constituted an *n* by 2 matrix of **Y**. **X** is a matrix full of 1s and **X**B simply represents the fixed effects other than SNP and the population structure. A is an 2 by 2 matrix of fixed effects of SNP, each row denoting effects of a particular genotype across two groups. **V** is a *k* by 2 matrix of population effects. **U** is an *n* by 2 matrix of unknown random effects and **E** is an *n* by 2 matrix of residual effects. **S** is an *n by 2* design matrix for the two genotypes of a SNP and **Q** is a *n* by *k* design matrix of 1s and 0s from results of fastStructure. Kinship matrix **K** is reflected as variance of random effects **U**. Because of the property with bivariate phenotype and the accompanying bivariate random effect, **U** was assumed to follow the multivariate distribution, with mean being a zero matrix, as shown in above equation. V${}_{g}$ is a 2 by 2 symmetric matrix of genetic variance component, V${}_{e}$ is a 2 by 2 symmetric matrix of environmental variance component, and MN${}_{n\times 2}$ (**0**, V${}_{1}$, V${}_{2}$) denotes the n t⁢i⁢m⁢e⁢s 2 matrix normal distribution with mean 0, row covariance matrix V${}_{1}$ (*n* by *n*), and column covariance matrix V${}_{2}$ (2 by 2). The first three parts including **X**B, **S**A, and **QV** are viewed as fixed effects, and **U** is viewed as random effects. In this manner, the *Q* + *K* model for GWAS of bivariate phenotype had been implemented, integrating both population structure effect (**Q**) and kinship effect (**K**). After model construction, GEMMA derived REMLE algorithm to solve *Vg* and *Ve* and other parameters in above equation. The threshold of GWAS was determined by 1000 permutation replicates. In each permutation, relationship between genotype and bivariate phenotype was reshuffled randomly. A minimum *P*-value from each permutation was selected out to construct an increasing vector of 1000 elements. By using 0.05 as the significance level, the 50th value was taken as the threshold for GWAS.

### Statistical Analysis

All statistical analyses were performed with SPSS v12.0 (SPSS Taiwan Corp., Taipei, Taiwan). *P <* 0.05 was considered statistically significant.

## Results

### Growth Plasticity of *S. aureus*

**FIGURE 1:**
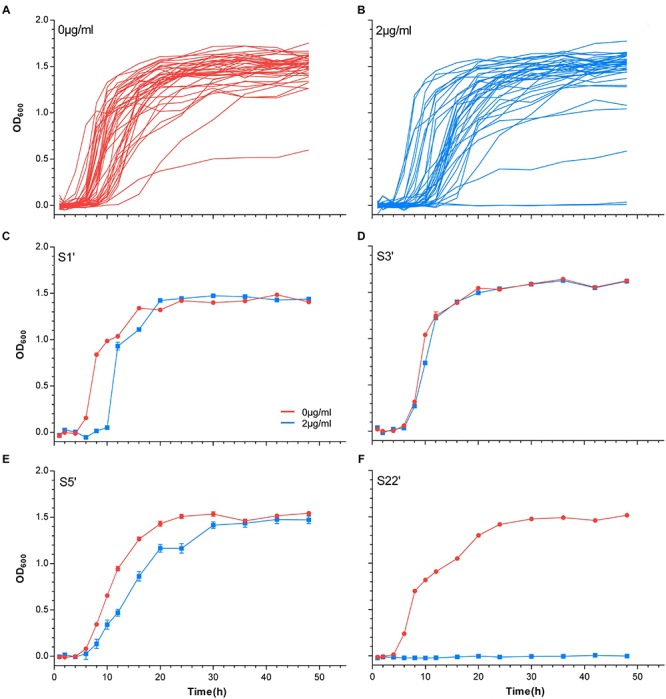
Growth curves of *S. aureus*. The growth curves of all strains at 0 μg/ml vancomycin culture **(A)** and 2 μg/ml vancomycin culture **(B)**. **(C–F)** The growth curves of S1’, S3’, S5’, and S22’ at 0 and 2 μg/ml.

The growth curves of untreated and 2 μg/ml vancomycin-treated cells were plotted to test the growth plasticity of *S. aureus* in vancomycin (Figures 1A,B). In total, 41 strains (S1’–S9’ and S11’–S42’) were obtained by 60-day *in vitro* vancomycin treatment in our previous study (Wang et al., 2016). In the absence of antibiotics, 40 *S. aureus* strains grew well, but S6’ grew slowly and had a modest exponential period. The maximum OD_600_ of S6’ reached 0.5 at 30 h, which was slightly lower than that of the other strains (Supplementary Figure S1). In the presence of 2 μg/ml of vancomycin, 13 isolates (S3’, S6’, S7’, S8’, S9’, S15’, S16’, S23’, S26’, S32’, S35’, S37’, and S39’) showed little or no difference compared to non-antibiotic conditions. The growth of thirteen strains (S5’, S11’, S13’, S17’, S19’, S20’, S27’, S31’, S33’, S34’, S38’, S40’, and S41’) in the exponential phase was slightly slower, but the time required to enter the exponential phase was not delayed. Twelve strains (S1’, S2’, S4’, S12’, S14’, S18’, S21’, S24’, S28’, S29’, S36’, and S42’) grew slow and took a longer time to reach the exponential phase. The other three strains (S22’, S25’, and S30’) with MIC ≥ 2 μg/ml did not grow on 2 μg/ml of vancomycin. This phenomenon might be caused by different culture conditions between MIC determination (on agar plate) and growth experiment (in liquid BHI medium). The growth curve of one strain from these four classes is shown in Figures 1C–F. The growth differences of the 41 strains revealed that they have varying degrees of growth fitness in response to vancomycin.

### MGRs in Different Cultures

**FIGURE 2:**
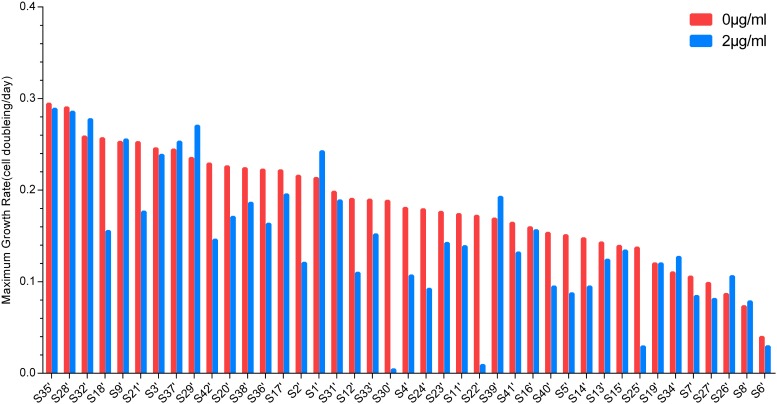
Maximum growth rates of 41 *S. aureus*. The maximum growth rates of strains in 0 and 2 μg/ml vancomycin treatments were sorted and compared.

**FIGURE 3:**
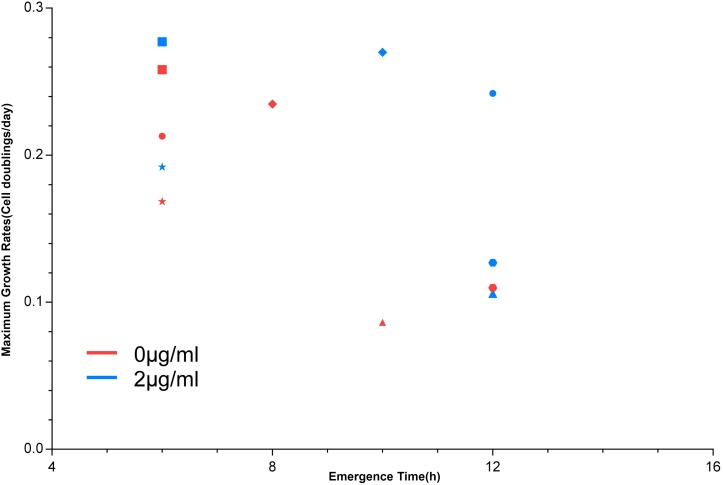
The emergence time of the maximum growth rate. The maximum growth rate emergence time of six strains in the growth process. Circle: S1’; triangle: S26’; rhombus: S29’; square: S32’; hexagon: S34’; pentagon: S39’.

The MGRs of 41 strains cultured with or without vancomycin were sorted. Figure 2 shows the MGR of S35’, which was the highest, and S6’, which was the lowest, in the non-vancomycin culture. The MGRs of S22’, S25’, and S30’ were dramatically decreased in the presence of vancomycin. In vancomycin group, the MGRs of nine strains (S2’, S4’, S5’, S12’, S14’, S18’, S24’, S40’, and S42’) were poor, and the MGRs of five strains (S2’, S4’, S5’, S12’, and S24’) were only approximately half of that without treatment. These 12 isolates with decreased MGRs in vancomycin group may exert poor growth plasticity, and be difficult to adapt changing environment. The MGRs of 13 strains (S6’, S7’, S11’, S13’, S17’, S20’, S21’, S23’, S27’, S33’, S36’, S38’, and S41’) were slightly lower in the presence of vancomycin. The other 16 strains (S1’, S3’, S8’, S9’, S15’, S16’, S19’, S26’, S28’, S29’, S31’, S32’, S34’, S35’, S37’, and S39’) showed no significant difference in MGR under different treatments, while six strains (S1’, S26’, S29’, S32’, S34’, and S39’) even had higher MGRs in vancomycin treatment. In antibiotic presence environment, the greater MGR indicates a better growth plasticity, which helps the strain to generate a larger population easily. The MGR emergence time could also reflect the growth plasticity of different strains. The MGRs and the MGR emergence time of S1’, S26’, S29’, S32’, S34’, and S39’ are listed in Figure 3. The MGRs of two strains (S32’ and S39’) appeared at 6 h and there was no delay following antibiotic treatment. S32’ showed a greater MGR than S39’. The MGR of S34’ occurred at 12 h with and without treatment. The MGRs of strains S26’ and S29’ were delayed for 2 h (from 8 to 10 h and from 10 to 12 h) in the presence of vancomycin. Combined with the growth curve data, these results suggest that the better growth adaption of S32’ is highly related to its growth plasticity.

### SNP Detection and Genetic Analysis

The sequencing statistics including average sequencing depth and coverage are shown in Supplementary Tables S2, S3. The average depth of sequencing was over 200 folds, and the coverage depth no less than 20X in whole genome region was the lowest at 88.93%. After mapping the reads to the reference genome, erroneous reads caused by polymerase chain reaction duplications (<1%) were removed. Finally, 61,869 high-quality SNPs with a *Q*-value ≥ 20 and enough supporting bases (≥4) were collected from 41 *S. aureus* strains. These SNPs covered the *S. aureus* genome by one per 46 bp. This SNP density should be sufficient for the identification of genomic regions in a GWAS. The clean sequence data reported in this study have been deposited in the Genome Sequence Archive in BIG Data Center (Big Data Center Members, 2018), Beijing Institute of Genomics (BIG), Chinese Academy of Sciences, under accession numbers CRA001125 that is publicly accessible at http://bigd.big.ac.cn/gsa.

To identify candidate genes that may be related to growth plasticity, a bivariate GWAS was employed to analyze the growth of 41 *S. aureus* strains. A total of 227 SNPs above the threshold value were identified from 14 time points. Manhattan plots of all time points are shown in Supplementary Figure S2. After screening, 51 positions that appeared at least twice were obtained and 15 SNPs were mapped to different annotated genes (Table 1 and Supplementary Table S4).

**TABLE 1:** Summary of candidate genes and proteins of 16 SNPs.

Position	Gene ID	Gene name	SNP	AA mutation	Frequency of positive SNPs	Annotation
264897	SAOUHSC_00246	—	T/G	I < - > I	24.39%	Drug transporter
382501	SAOUHSC_00375	*guaA*	G/A	E < - > E	51.22%	GMP synthase
603363	SAOUHSC_00611	*argS*	T/A	T < - > T	53.66%	Arginyl-tRNA synthetase
615798	SAOUHSC_00625	*mnhA2*	T/C	I < - > I	39.02%	Putative monovalent cation/H+ antiporter subunit A
738836	non-coding region	—	T/C	/	39.02%	Non-coding region between SAOUHSC_00755 and *glxK* (SAOUHSC_00756)
906471	SAOUHSC_00933	*trpS*	G/A	L < - > L	58.54%	Tryptophanyl-tRNA synthetase
965494	SAOUHSC_00994	*atl*	C/T	K < - > K	43.90%	Bifunctional autolysin
1307807	SAOUHSC_01364	*tyrA*	G/T	L < - > M	34.15%	Prephenate dehydrogenase
1394043	SAOUHSC_01447	*ebh*	G/A	N < - > N	80.49%	Extracellular matrix-binding protein
1612045	SAOUHSC_01705	—	C/T	A < - > A	51.22%	Enterotoxin family protein
1775112	SAOUHSC_01868	*pepV*	G/A	N < - > N	51.22%	Dipeptidase PepV
1842166	SAOUHSC_01933	*hsdM*	T/G	Y < - > S	31.71%	Type I restriction-modification system subunit M
2290791	SAOUHSC_02467	*alsD*	A/G	F < - > F	63.42%	Alpha-acetolactate decarboxylase
2535773	SAOUHSC_02760	*gltB*	C/T	T < - > T	73.17%	Glutamate synthase subunit alpha
2588313	SAOUHSC_02809	*gntR*	A/G	P < - > P	41.46%	Gluconate operon transcriptional repressor
2621187	SAOUHSC_02848	*glcB*	C/T	A < - > A	43.90%	PTS system glucose-specific transporter subunit IIABC

Position 264897 is located in SAOUHSC_00246, which is related to drug transporters. *GuaA* (SNP 382501) encodes GMP synthetase. GMP synthase catalyzes the generation of GMP by *de novo* synthesis. The *argS* gene(SNP 603363) is involved in arginine-tRNA aminoacylation. The *mnhA2* gene at position 615798 is related to the transmembrane transport of ions. Position 906471 of *trpS* is related to the synthesis of tryptophanyl-tRNA synthetase. SNP 965494, which was significant at time points 2, 3, 4, 5, 6, and 7, is located in *atl* gene, which is involved in peptidoglycan decomposition and the activation of amidase. The *tyrA* gene (SNP 1307807) encodes prephenate dehydrogenase. SNP 1394043 is located in *ebh* (SAOUHSC_01447), which edcodes extracellular matrix-binding protein. Position 1612045 is located in SAOUHSC_01705, which encodes enterotoxin family proteins. Position 1775112 (*pepV*) encodes the dipeptidase. *AlsD* (SNP 2290791) and *gltB* (SNP 2535773) are involved in acetoin and glutamate biosynthesis, respectively. The *hsdM* (SNP 1842166) encodes the type I restriction-modification system subunit M. *GntR* (SNP 2588313) and *glcB* (SNP 2621187) are related to gluconate transcription and glucose transmembrane transport, respectively.

**FIGURE 4:**
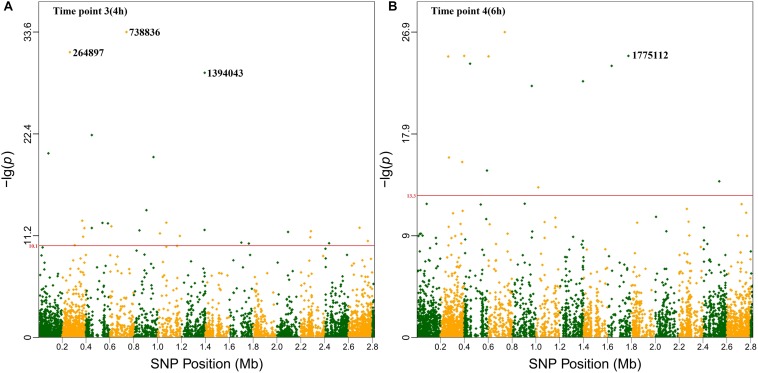
Manhattan plots of the four significant SNPs The *x*-axis showed SNP positions (Mb) and the *y*-axis was the –log*P*-value resulting from the association test. Each dot in the plot represented an SNP, and a reference line was used on the *y*-axis to reflect genome-wide significance. **(A)** Time point 3 (4 h). **(B)** Time point 4 (6 h).

**FIGURE 5:**
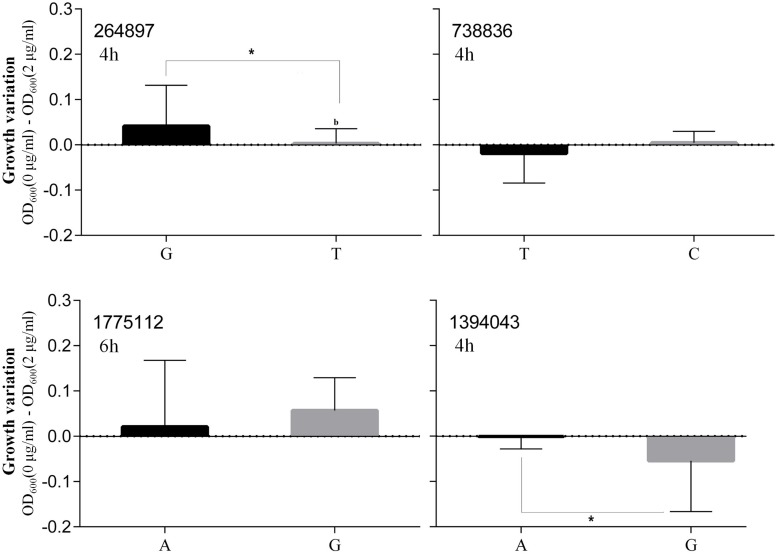
Differences of the growth of two genotypes The *x*-axis represented two genotypes and the *y*-axis showed the growth variation between the two cultures.

**FIGURE 6:**
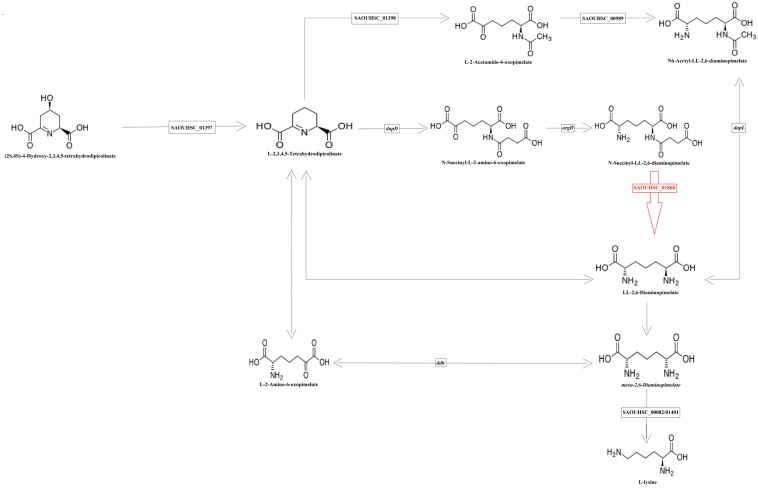
Schematic overview of the partial reaction of lysine formation The enzyme encoded by the SAOUHSC_01868 (in red) regulates the production of lysine.

Based on the highest –log*P*-value and the frequency of occurrence (Supplementary Table S4), we focused on four significant SNPs (one non-coding region and three annotated proteins) (Figure 4), and the relationship between growth plasticity and genotype was analyzed. The growth variation between the two cultures was calculated by subtracting the OD_600_ at 2 μg/ml (treated) from that at 0 μg/ml (untreated), and the strains were divided into two groups according to the genotype of each SNP (Figure 5). Position 738836, with the highest –log*P*-value (33.60515) at 4 h, is located in a non-coding region between SAOUHSC_00755 and *glxK* (SAOUHSC_00756). Based on the comparison of the two genotypes, the growth plasticity was significantly different between genotypes T and C (Figure 5). Position 1394043 was the most significant locus of extracellular matrix-binding protein (*ebh*, SAOUHSC_01447), with a –log*P*-value of 29.12073 at 4 h. Except for 1394043, four additional high-frequency SNPs (SNP 1378107, 1392993, 1393088, and 1401382) were also mapped to *ebh* gene (Supplementary Table S4). The growth variation in two bases represented distinctly different trends (*P* = 0.0149), and genotype A showed better growth plasticity (Figure 5). The growth of two genotypes at SNP 264897 (SAOUHSC_00246) was lower in the presence of vancomycin, but genotype T showed better adaption to the vancomycin culture than genotype G (*P* = 0.0494) (Figure 5). Regarding position 1775112, the growth of genotype A was better than that of C under antibiotic pressure, although the difference was not significant (*P* = 0.3279) (Figure 5). Position 1775112 is located in *pepV* (SAOUHSC_01868), which encodes the dipeptidase. A schematic model of the lysine-signaling pathway mediated by SAOUHSC_01868 discovered in proteomic studies is shown in Figure 6. *PepV* regulates lysine synthesis, and lysine affected *S. aureus* growth in calf serum. These results indicate that non-coding region (SNP 738836), *ebh* (SNP 1394043), drug transporter (SNP 264897), and *pepV* (SNP 1775112) are important for the growth plasticity of *S. aureus* in vancomycin culture.

## Discussion

Phenotypic changes include behavior, physiology, morphology, and growth, which can be found on individuals or across generations (Tali et al., 2012). Phenotypic plasticity reflects the relationship between an individual and its environment. Despite the great effects of microorganisms on the environment, studies of unicellular organisms, especially bacteria, are insufficient. In this study, we investigated the relationship between growth plasticity and drug resistance in *S. aureus*. In total, 41 strains, including 29 VISA strains, were cultivated in the absence or presence of vancomycin. The growth plasticity was analyzed by a bivariate GWAS and several significant SNPs were identified. Our results suggest that four SNPs (non-coding region between SAOUHSC_00755 and *glxK*, *ebh*, drug transporter, and *pepV*) may play important roles in the growth plasticity of *S. aureus*.

As the result of interactions between genotype and environment, the phenotypes of an individual can vary (Fusco and Minelli, 2010). The ability of a given genotype to develop different phenotypes helps it survive in different physical and social environments (Nadeau et al., 2017). Individual behavior adjustments to climate change are conducive to rapid adaptation of the population to environmental changes (Charmantier et al., 2008). Even simple bacteria can respond to environmental changes and produce phenotypic plasticity (Kummerli et al., 2009). In yeast, when the population was in a stable environment, the individual had a larger impact and consistent plasticity and all alleles in a high-variance environment developed high plasticity (Yadav et al., 2016). Antibiotic-resistant *S. aureus* is a major threat to public health (Dantes et al., 2013). Previous studies have suggested that some *rpoB* mutations in rifampicin-resistant *S. aureus* enhance the clinical relevance of phenotypic plasticity, such as promoting persistent infection and decreasing sensitivity (Baek et al., 2015; Romain et al., 2017). The evolution of resistant strains depends on both genetic variation and phenotypic plasticity. It is easy to envision that a strain with better growth plasticity could generate a larger population and develop VISA more easily in a vancomycin environment. Although several genes and mutations are responsible for VISA development, the relationship between growth plasticity and vancomycin resistance in *S. aureus* remains unknown. In our growth plasticity experiment, 41 *S. aureus* strains were grown in the absence or presence of vancomycin for 48 h. The OD_600_ values were collected at 14 time points to represent bacterial growth. SNPs that were significantly related to growth plasticity were identified by a bivariate GWAS. Our results suggest that under vancomycin pressure, growth plasticity varies in different strains even with the same MIC, and there is no direct relationship between the MIC and growth rate (data not shown).

The successful publication of a human GWAS in 2005 offered a new method for gene discovery and the study of complex biological process (Klein et al., 2005). In recent years, GWAS have successfully identified thousands of SNPs. The emergence of a new generation of sequencing technology is conducive to a more comprehensive understanding of microorganisms (Forde and O’Toole, 2013). This new method, which captures lineage-level associations, has greatly promoted the development of microbial GWAS (Earle et al., 2016). The generation of genomic data makes it easier to distinguish genetic differences among different strains with the same phenotype to more accurately predict evolutionary trends (Koser et al., 2012). Genome-wide sequencing revealed that the evolution of specific genotypes of *Escherichia coli* enhanced tolerance to isobutanol (Minty et al., 2011). Bacterial GWAS will not only expand our knowledge of microorganisms, but also detect the environmental responses of bacteria (Daniel, 2016). Until now, most bacterial GWAS have used a single phenotype to examine the relationship between antibiotic resistance and bacterial genes (Power et al., 2016). The emergence of bivariate GWAS could reduce false-positives and offer greater stability (Goudey et al., 2013; Justin et al., 2013). Previous studies have identified genes that regulate osteoporosis in humans by using bone mineral density and alcohol intake as phenotypes (Lu et al., 2017). The genetic relationship between lipids and inflammation was made much clearer by a bivariate GWAS (Ligthart et al., 2016). However, bivariate GWAS has not yet been applied to bacterial research.

In our study, 227 significant SNPs related to the growth variation between cultures in the absence or presence of vancomycin were identified by a bivariate GWAS. As shown in Supplementary Figure S2, the majority of significant SNPs were found at time points 6–8, which represent the end of exponential growth phase of most strains. There are also many significant SNPs at time points 2 and 3, which represent the early stage of exponential phase or the end of lag phase. These results indicate that the exponential phase is highly associated with growth plasticity of bacteria. Fifteen high-frequency SNPs were mapped to different annotated genes. Most genes are involved in bacterial growth and are related to growth plasticity. In our previous study, numbers of mutation sites were observed in six drug-resistance genes in these strains (Wang et al., 2016). In this study, growth experiment was focused on the phenotypic plasticity of *S. aureus* in different cultural conditions. It is reasonable that many significant SNPs are not located in drug resistance genes, which indicate that vancomycin resistance and growth plasticity might be affected by different pathways. Based on the *P*-values and frequency of occurrence, we focused on four loci (SNPs 738836, 1394043, 264897, and 1775112), which may be crucial for the growth plasticity of *S. aureus*. The most significant SNP 738836 is located in a non-coding region. Although non-coding regions do not encode proteins, they still regulate gene expression in various biological processes of bacteria (Luay et al., 2010). SNP 738836 was mapped between SAOUHSC_00755 and *glxK* (SAOUHSC_00756). According to a previous report, SAOUHSC_00755 may be related to eDNA release, which promotes biofilm formation (DeFrancesco et al., 2017). During the formation of biofilm, the releasing of eDNA represents that the cell membrane synthesis reaches its peak (Mann et al., 2009). The release of eDNA promotes the formation of biofilm, which helps the strain to adapt changing environment. Our results indicate that SNP 738836 might affect cell membrane synthesis and play an important regulatory role in the growth plasticity of *S. aureus* in vancomycin. The growth of SNP 1394043 was remarkably different (as determined by a *t*-test of the A and G genotypes). *Ebh* (SNP 1394043) contributes to cell membrane synthesis and envelope assembly pathways (Clarke et al., 2002). It has been found that the sensitivity of *S. aureus* to methicillin increases after *ebh* mutation, and the toxicity in mice decreases (Cheng et al., 2014). In this study, different genotypes of *ebh* may affect peptidoglycan synthesis and the adaption to the antibiotic environment. Drug transporter (SNP 264897) encodes a putative transmembrane efflux pump protein, shows 79% similarity with *norB* (Truong-Bolduc et al., 2017). Previous study confirms that *norB* encodes multidrug resistance efflux pumps and is related to the ciprofloxacin resistance of MRSA (Kwak et al., 2013). Bacteria can pump intracellular antimicrobial drugs out of the cell through an efflux pump system, thus reducing the concentration of drugs in the bacteria and leading to bacterial growth (Sun et al., 2014). As shown in Figure 6, SOUHSC_01868 (*pepV*) of SNP 1775112 is an important step in the synthesis of lysine and regulates synthesis downstream of lysine. This protein also catalyzes the conversion of *N*-succinyl-L,L-2,6-diaminopimelate to L,L-2,6-diaminopimelate and ultimately yields lysine and other intermediate products that indirectly affect peptidoglycan biosynthesis and cell growth (Niven et al., 1973). In summary, bivariate GWAS has high accuracy in the analysis of phenotypic plasticity, and the four SNPs identified herein may greatly influence the phenotypic plasticity of *S. aureus*.

## Conclusion

We chose vancomycin as an environmental stress factor to study the phenotypic plasticity of *S. aureus*. A bivariate GWAS was used to identify potential SNPs and genes responsible for growth plasticity. Our results provide an alternative strategy to dissect the adaptive growth of *S. aureus* in an antibiotic environment and highlight the feasibility of bivariate GWAS in the phenotypic plasticity research of bacteria.

## Author Contributions

YJ and XH conceived and designed the experiments. MR, XZ, and JB performed the experiments. MY, XX, and YJ analyzed the data. MR and YJ wrote the manuscript. All authors reviewed the manuscript.

## Conflict of Interest Statement

The authors declare that the research was conducted in the absence of any commercial or financial relationships that could be construed as a potential conflict of interest.

## Supplementary Material

FIGURE S1 Growth curves of 41 strains. The growth curves of all strains at two treatments.

FIGURE S2 Manhattan plots of significant SNPs. The Manhattan plots of all time points.

TABLE S1 All strains’ background.

TABLE S2 Sequencing data quality summary.

TABLE S3 Reads and reference sequence alignment.

TABLE S4 Summary of 51 SNPs.
