# Supplementary figures and images for: Phenotypic Plasticity of Staphylococcus aureus in Liquid Medium Containing Vancomycin
Source: Front Microbiol. 2019 Apr 16;10:809. doi: 10.3389/fmicb.2019.00809 (PMC6477096; doi:10.3389/fmicb.2019.00809)

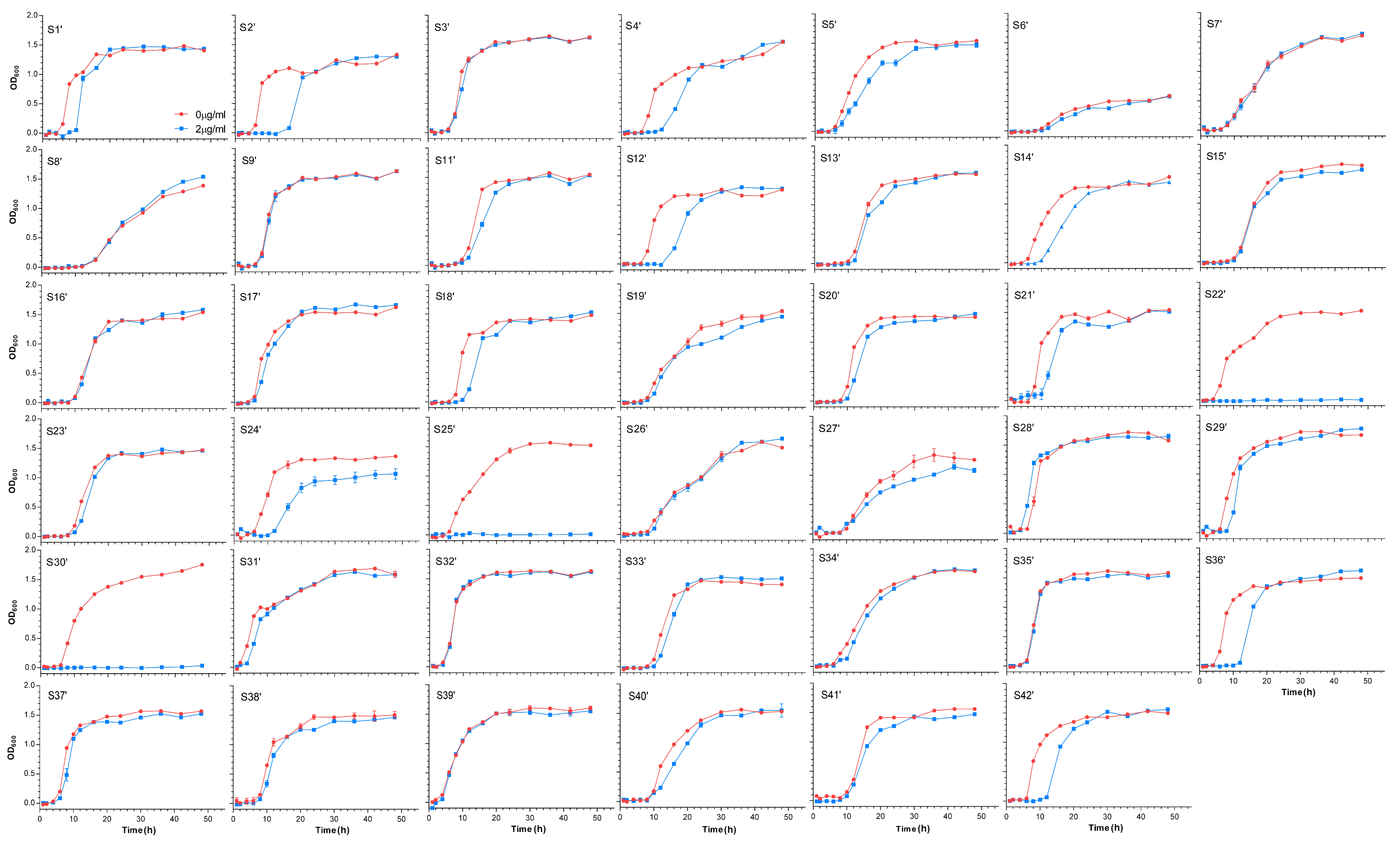

Supplement: FIGURE S1 — Growth curves of 41 strains. The growth curves of all strains at two treatments. [file Image_1.JPEG]

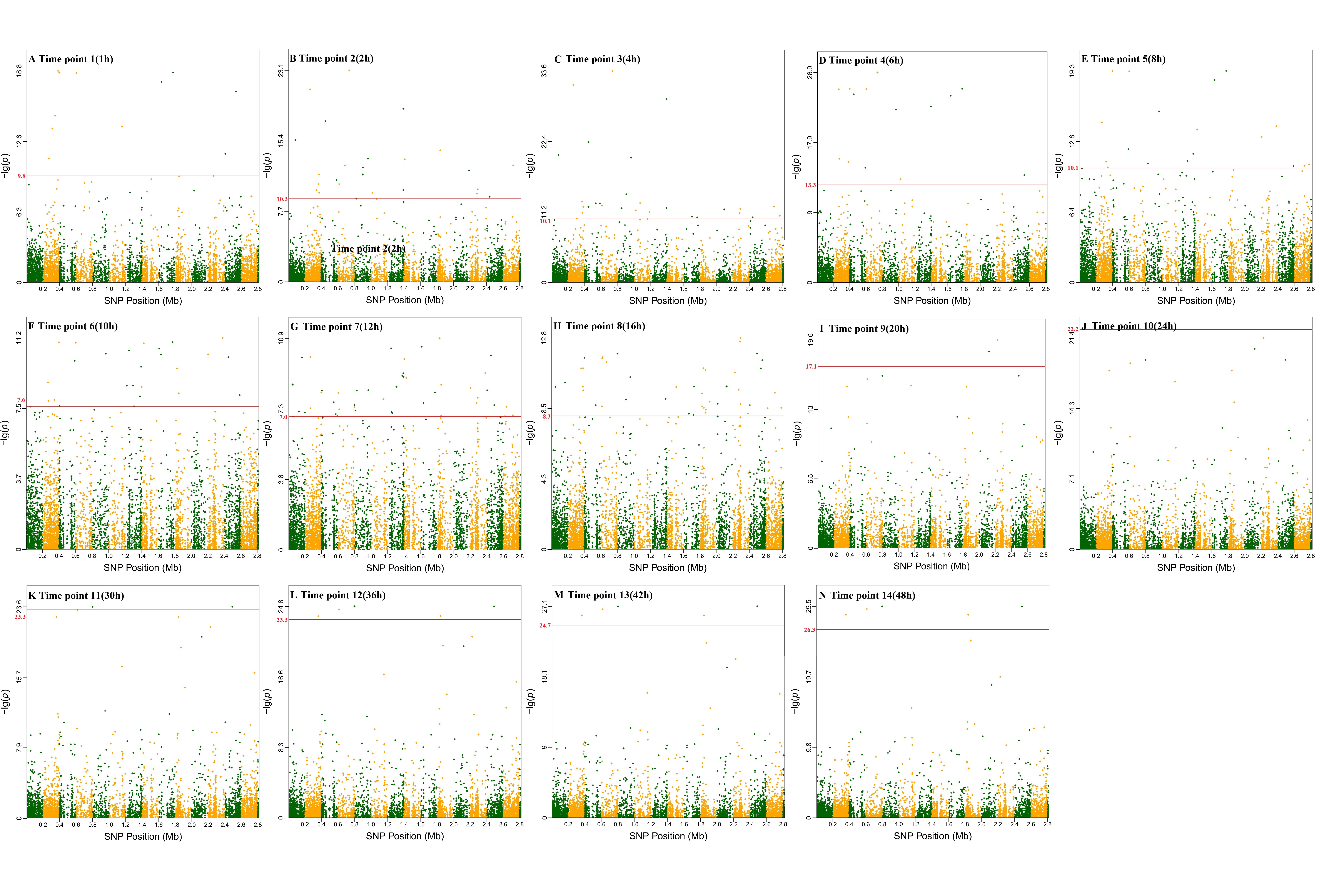

Supplement: FIGURE S2 — Manhattan plots of significant SNPs. The Manhattan plots of all time points. [file Image_2.JPEG]
