# Supplementary material for: Phenotypic Plasticity of Staphylococcus aureus in Liquid Medium Containing Vancomycin
Source: Front Microbiol. 2019 Apr 16;10:809. doi: 10.3389/fmicb.2019.00809 (PMC6477096; doi:10.3389/fmicb.2019.00809)
Supplement: TABLE S1 — All strains’ background. [file Table_1.DOCX]

Supplementary Table S1 All strains’ background

| Strain | Provider | ST | MIC(µg/mL) | OXA/RIF/TEI | Accession number |
| --- | --- | --- | --- | --- | --- |
| S1’^a^ (S1^b)^ | CICC (21676^c^) | 8^d^ | 10^e^ | S/S/S^f^ | CRX029816^g^ |
| S2’(S2) | CICC(21600) | 5 | 3 | S/S/S | CRX029817 |
| S3’(S3) | ACCC(01334) | 239 | 10 | R/R/S | CRX029818 |
| S4’(S4) | ACCC(01340) | 243 | 3 | S/S/S | CRX029819 |
| S5’(S5) | ACCC(01332) | 239 | 4 | S/S/S | CRX029820 |
| S6’(S6) | ACCC(01331) | 243 | 3 | S/S/S | CRX029821 |
| S7’(S7) | ACCC(01339) | 243 | 10 | S/S/S | CRX029822 |
| S8’(S8) | CFCC(10341) | 6 | 16 | S/S/I | CRX029823 |
| S9’(S9) | CGMCC(1.8721) | 5 | 12 | S/S/I | CRX029824 |
| S11’(S11) | CGMCC(1.1476) | 96 | 8 | S/S/S | CRX029825 |
| S12’(S12) | CPCC(141396) | 464 | 3 | S/S/S | CRX029826 |
| S13’(S13) | CPCC(140594) | 464 | 4 | S/S/S | CRX029827 |
| S14’(S14) | CPCC(140575) | 464 | 3 | S/S/S | CRX029828 |
| S15’(S15) | CICC(21648) | 97 | 16 | S/R/I | CRX029829 |
| S16’(S16) | CICC(10786) | 239 | 16 | S/R/I | CRX029830 |
| S17’(S17) | CICC(22942) | 943 | 4 | S/S/S | CRX029831 |
| S18’(S18) | CCTCC(AB 94004) | 96 | 3 | S/S/S | CRX029832 |
| S19’(S19) | CCTCC(AB 91093) | 464 | 4 | S/S/S | CRX029833 |
| S20’(S20) | CCTCC(AB 91053) | 464 | 4 | S/S/S | CRX029834 |
| S21’(S21) | CICC(23699) | 464 | 4 | S/S/S | CRX029835 |
| S22’(S22) | CICC(23656) | 243 | 3 | S/S/S | CRX029836 |
| S23’(S23) | CICC(22944) | 943 | 12 | S/S/S | CRX029837 |
| S24’(S24) | CGMCC(1.2465) | 464 | 3 | S/S/S | CRX029838 |
| S25’(S25) | CCTCC(AB 91119) | 243 | 4 | S/S/S | CRX029839 |
| S26’(S26) | CICC(10201) | 464 | 4 | S/S/S | CRX029840 |
| S27’(S27) | ACCC(10499) | 464 | 8 | S/S/S | CRX029841 |
| S28’(S28) | ACCC(01012) | 943 | 14 | S/S/I | CRX029842 |
| S29’(S29) | CPCC(141405) | 464 | 3 | S/S/S | CRX029843 |
| S30’(S30) | CMCC(26003) | 464 | 3 | S/S/S | CRX029844 |
| S31’(S31) | CMCC(26112) | 464 | 4 | S/S/S | CRX029845 |
| S32’(S32) | ACCC(01011) | 243 | 12 | S/S/I | CRX029846 |
| S33’(S33) | CMCC(26001) | 30 | 3 | S/S/S | CRX029847 |
| S34’(S34) | CPCC(141431) | 464 | 6 | R/S/S | CRX029848 |
| S35’(S35) | CPCC(140660) | 8 | 12 | S/R/S | CRX029849 |
| S36’(S36) | CGMCC(1.1529) | 770 | 3 | S/S/S | CRX029850 |
| S37’(S37) | CAU(P1) | 9 | 8 | R/R/S | CRX029851 |
| S38’(S38) | CAU(AB18) | 9 | 8 | S/S/S | CRX029852 |
| S39’(S39) | CAU(CD1) | 9 | 8 | S/S/S | CRX029853 |
| S40’(S40) | CAU(CD9) | 9 | 8 | S/S/S | CRX029854 |
| S41’(S41) | CAU(CD7) | 9 | 16 | S/S/S | CRX029855 |
| S42’(S42) | ACCC(01336) | 5 | 4 | S/S/S | CRX029856 |

^a^ Strain number in this study; ^b^ Parental strain number in previous study (Wang et al., 2016); ^c^ Center ID of the parental strain; ^d^ ST results of parental strains, ^e^ MIC of the Strain after vancomycin treatment for 60 days in vitro; ^f^ OXA/RIF/TEI: Oxacillin/Rifampicin/Teicoplanin S: susceptible; I: intermediate; R: resistant,

^g^ Accession number of sequencing data in BIG Data Center (http://bigd.big.ac.cn)

CGMCC: China General Microbiological Culture Collection Center

CFCC: China Forestry Culture Collection Center

CICC: China Center of Industrial Culture Collection

CMCC: National Center for Medical Culture Collections

ACCC: Agricultural Culture Collection of China

CCTCC: China Center for Type Culture Collection

CPCC: China Pharmaceutical Culture Collection

CAU: China Agricultural University
