# Supplementary material for: Phenotypic Plasticity of Staphylococcus aureus in Liquid Medium Containing Vancomycin
Source: Front Microbiol. 2019 Apr 16;10:809. doi: 10.3389/fmicb.2019.00809 (PMC6477096; doi:10.3389/fmicb.2019.00809)
Supplement: TABLE S2 — Sequencing data quality summary. [file Table_2.DOCX]

Supplementary Table S2 Sequencing data quality summary

| Sample | Raw Base(bp) | Clean Base(bp) | Effective Rate(%) | Error Rate(%) | Q20(%) | Q30(%) | GC Content(%) |
| --- | --- | --- | --- | --- | --- | --- | --- |
| S1’ | 1,276,220,100 | 1,234,355,700 | 96.72 | 0.01 | 97.34 | 93.47 | 32.76 |
| S2’ | 1,207,527,600 | 1,164,902,400 | 96.47 | 0.01 | 97.34 | 93.45 | 32.58 |
| S3’ | 1,097,338,500 | 1,054,156,500 | 96.06 | 0.01 | 97.24 | 93.25 | 32.95 |
| S4’ | 1,024,674,000 | 996,435,000 | 97.24 | 0.01 | 97.58 | 93.9 | 32.89 |
| S5’ | 1,005,031,500 | 947,010,600 | 94.23 | 0.01 | 96.06 | 90.91 | 33.4 |
| S6’ | 982,932,600 | 941,070,000 | 95.74 | 0.01 | 96.9 | 92.61 | 32.8 |
| S7’ | 1,027,182,000 | 977,821,200 | 95.19 | 0.01 | 96.73 | 92.27 | 33.28 |
| S8’ | 1,158,221,700 | 1,097,237,700 | 94.73 | 0.02 | 96.34 | 91.58 | 33.14 |
| S9’ | 1,237,908,900 | 1,170,174,300 | 94.53 | 0.02 | 96.24 | 91.35 | 32.33 |
| S11’ | 1,080,319,800 | 1,062,825,600 | 98.38 | 0.01 | 97.88 | 94.43 | 32.62 |
| S12’ | 1,331,588,100 | 1,311,063,600 | 98.46 | 0.01 | 97.98 | 94.64 | 32.78 |
| S13’ | 1,098,426,600 | 1,080,812,100 | 98.4 | 0.01 | 97.99 | 94.65 | 32.93 |
| S14’ | 977,382,900 | 960,207,600 | 98.24 | 0.01 | 98.04 | 94.77 | 32.77 |
| S15’ | 1,153,930,800 | 1,132,962,300 | 98.18 | 0.01 | 98.06 | 94.8 | 32.73 |
| S16’ | 1,292,799,000 | 1,267,027,200 | 98.01 | 0.01 | 98.1 | 94.92 | 32.75 |
| S17’ | 1,103,493,900 | 1,083,543,900 | 98.19 | 0.01 | 98.06 | 94.83 | 32.83 |
| S18’ | 1,101,992,400 | 1,069,692,900 | 97.07 | 0.01 | 97.96 | 94.6 | 33.14 |
| S19’ | 1,172,319,900 | 1,128,285,000 | 96.24 | 0.01 | 97 | 92.72 | 32.87 |
| S20’ | 2,582,253,600 | 2,508,086,700 | 97.13 | 0.01 | 97.91 | 94.82 | 33.37 |
| S21’ | 1,154,775,300 | 1,114,807,200 | 96.54 | 0.01 | 97.9 | 94.45 | 33.32 |
| S22’ | 1,005,323,100 | 944,565,900 | 93.96 | 0.02 | 95.95 | 90.75 | 33.28 |
| S23’ | 1,417,425,600 | 1,363,396,500 | 96.19 | 0.01 | 96.32 | 91.38 | 32.88 |
| S24’ | 2,658,411,600 | 2,581,080,000 | 97.09 | 0.01 | 97.84 | 94.66 | 33.35 |
| S25’ | 1,187,688,600 | 1,141,178,100 | 96.08 | 0.01 | 97.14 | 93.05 | 33.09 |
| S26’ | 1,088,412,900 | 1,053,000,300 | 96.75 | 0.01 | 97.22 | 93.23 | 33.21 |
| S27’ | 1,427,494,500 | 1,378,908,300 | 96.6 | 0.01 | 96.51 | 91.74 | 32.92 |
| S28’ | 1,184,597,400 | 1,140,968,400 | 96.32 | 0.01 | 96.94 | 92.67 | 33.02 |
| S29’ | 1,216,412,700 | 1,173,562,200 | 96.48 | 0.01 | 97.19 | 93.14 | 32.81 |
| S30’ | 1,055,945,100 | 997,863,000 | 94.5 | 0.02 | 96.28 | 91.43 | 33.39 |
| S31’ | 1,103,836,500 | 1,049,487,600 | 95.08 | 0.01 | 96.51 | 91.85 | 32.7 |
| S32’ | 1,094,899,500 | 1,054,940,400 | 96.35 | 0.01 | 97.13 | 93.06 | 33.15 |
| S33’ | 988,878,600 | 948,252,600 | 95.89 | 0.01 | 96.84 | 92.44 | 33.06 |
| S34’ | 1,087,321,200 | 1,064,582,100 | 97.91 | 0.01 | 97.63 | 93.98 | 32.57 |
| S35’ | 1,220,067,000 | 1,192,517,100 | 97.74 | 0.01 | 97.56 | 93.84 | 32.62 |
| S36’ | 1,033,618,500 | 982,873,500 | 95.09 | 0.01 | 96.63 | 92.11 | 32.81 |
| S37’ | 2,619,620,700 | 2,332,630,800 | 89.04 | 0.01 | 97.18 | 93.37 | 33.42 |
| S38’ | 1,001,803,800 | 949,707,300 | 94.8 | 0.01 | 96.53 | 91.9 | 32.95 |
| S39’ | 1,032,318,900 | 972,514,800 | 94.21 | 0.02 | 96.56 | 92.03 | 33.2 |
| S40’ | 1,143,993,600 | 1,093,744,200 | 95.61 | 0.01 | 96.8 | 92.39 | 33.09 |
| S41’ | 1,254,948,600 | 1,209,438,900 | 96.37 | 0.01 | 97.13 | 93.06 | 33.06 |
| S42’ | 991,215,300 | 960,145,800 | 96.87 | 0.01 | 97.37 | 93.5 | 32.25 |

Sample: Strain number

Raw Base: The output of raw data, the number of sequencing sequences multiplied by the length of the sequence, in bp units.

Clean Base: The amount of valid data after filtering, the number of sequencing sequences after filtering multiplied by the length of sequencing sequence, in bp units

Effective Rate: Get the ratio of clean data to raw data after filtering

Error Rate: Sequencing error rate, calculated by formula Qphred=-10 log10(e)

Q20: Number percentage of bases with correct recognition rate of more than 99%.

Q30: Number percentage of bases with correct recognition rate of 99.9% or more

GC Content: The total number of bases G and C as a percentage of the total number of bases
