# Supplementary material for: Phenotypic Plasticity of Staphylococcus aureus in Liquid Medium Containing Vancomycin
Source: Front Microbiol. 2019 Apr 16;10:809. doi: 10.3389/fmicb.2019.00809 (PMC6477096; doi:10.3389/fmicb.2019.00809)
Supplement: TABLE S3 — Reads and reference sequence alignment. [file Table_3.DOCX]

Supplementary TableS3 Reads and reference sequence alignment

| Sample | Mapped reads | Total reads | Mapping rate(%) | Average depth(X) | Coverage 20X(%) |
| --- | --- | --- | --- | --- | --- |
| S1’ | 7,382,267 | 8,229,038 | 89.71 | 310.88 | 96.38 |
| S2’ | 6,262,497 | 7,766,016 | 80.64 | 266.81 | 94.73 |
| S3’ | 5,927,812 | 7,027,710 | 84.35 | 266.87 | 91.38 |
| S4’ | 5,921,076 | 6,642,900 | 89.13 | 266.42 | 89.21 |
| S5’ | 5,448,061 | 6,313,404 | 86.29 | 258.01 | 90.57 |
| S6’ | 5,617,228 | 6,273,800 | 89.53 | 254.63 | 90.59 |
| S7’ | 5,845,224 | 6,518,808 | 89.67 | 260.91 | 90.61 |
| S8’ | 6,428,237 | 7,314,918 | 87.88 | 275.54 | 94.69 |
| S9’ | 5,966,937 | 7,801,162 | 76.49 | 256.82 | 93.92 |
| S11’ | 6,491,319 | 7,085,504 | 91.61 | 311.48 | 93.25 |
| S12’ | 8,222,586 | 8,740,424 | 94.08 | 380.47 | 93.34 |
| S13’ | 6,853,124 | 7,205,414 | 95.11 | 318.57 | 93.27 |
| S14’ | 5,991,961 | 6,401,384 | 93.6 | 284.91 | 93.29 |
| S15’ | 6,893,549 | 7,553,082 | 91.27 | 327.43 | 93.52 |
| S16’ | 7,405,335 | 8,446,848 | 87.67 | 338.2 | 93.54 |
| S17’ | 6,453,867 | 7,223,626 | 89.34 | 301.54 | 92.70 |
| S18’ | 6,507,015 | 7,131,286 | 91.25 | 306.39 | 93.24 |
| S19’ | 7,082,944 | 7,521,900 | 94.16 | 319.7 | 93.40 |
| S20’ | 15,989,549 | 16,720,578 | 95.63 | 682.25 | 93.37 |
| S21’ | 6,994,291 | 7,432,048 | 94.11 | 334.22 | 93.34 |
| S22’ | 5,357,872 | 6,297,106 | 85.08 | 240.28 | 90.62 |
| S23’ | 6,721,276 | 9,089,310 | 73.95 | 304.99 | 92.80 |
| S24’ | 16,117,955 | 17,207,200 | 93.67 | 698.14 | 93.42 |
| S25’ | 6,599,709 | 7,607,854 | 86.75 | 289.14 | 90.66 |
| S26’ | 6,694,558 | 7,020,002 | 95.36 | 290.09 | 93.35 |
| S27’ | 7,556,391 | 9,192,722 | 82.2 | 334.16 | 93.38 |
| S28’ | 6,599,053 | 7,606,456 | 86.76 | 293.69 | 92.78 |
| S29’ | 7,408,680 | 7,823,748 | 94.69 | 329.38 | 93.37 |
| S30’ | 6,236,589 | 6,652,420 | 93.75 | 278.06 | 93.34 |
| S31’ | 5,766,152 | 6,996,584 | 82.41 | 258.29 | 90.68 |
| S32’ | 6,164,318 | 7,032,936 | 87.65 | 270.07 | 90.64 |
| S33’ | 5,504,153 | 6,321,684 | 87.07 | 244.52 | 93.35 |
| S34’ | 5,695,393 | 7,097,214 | 80.25 | 255.23 | 92.40 |
| S35’ | 7,019,869 | 7,950,114 | 88.3 | 313.25 | 93.58 |
| S36’ | 6,175,491 | 6,552,490 | 94.25 | 279.8 | 91.27 |
| S37’ | 13,534,637 | 15,550,872 | 87.03 | 636.77 | 89.24 |
| S38’ | 5,179,634 | 6,331,382 | 81.81 | 232.42 | 91.38 |
| S39’ | 5,759,571 | 6,483,432 | 88.84 | 243.55 | 88.93 |
| S40’ | 5,413,764 | 7,291,628 | 74.25 | 239.38 | 92.32 |
| S41’ | 6,544,823 | 8,062,926 | 81.17 | 283.18 | 93.61 |
| S42’ | 4,601,587 | 6,400,972 | 71.89 | 200.03 | 94.63 |

Sample: Strain number

Mapped reads: the total reads comparing on the reference genome

Total reads: the total number of reads for valid sequencing data

Mapping rate: the number of reads compared to the reference genome divided by the number of reads for valid sequencing data

Average depth: average sequencing depth, comparing the total number of bases of the reference genome divided by genome size

Coverage 20X: Ratio of base coverage depth not less than 20X in whole genome region
