# Supplementary material for: Phenotypic Plasticity of Staphylococcus aureus in Liquid Medium Containing Vancomycin
Source: Front Microbiol. 2019 Apr 16;10:809. doi: 10.3389/fmicb.2019.00809 (PMC6477096; doi:10.3389/fmicb.2019.00809)
Supplement: TABLE S4 — Summary of 51 SNPs. [file Table_4.DOCX]

Supplementary TableS4 Summary of 51 SNPs

| Posintion | -log*p* | Gene ID | Gene name | SNP | AA mutation | Time points | Annotation |
| --- | --- | --- | --- | --- | --- | --- | --- |
| 738836 | 33.6052 | / | / | T<->C | / | 2/3/4 | non-coding region between SAOUHSC_00755 and *glxK* |
| 264897 | 31.395 | SAOUHSC_00246 | — | T<->G | I<->I | 2/3/4 | drug transporter |
| 800827 | 29.4901 | / | / | T<->C | / | 7/8/11/12/13/14 | non-coding region between SAOUHSC_00818 and 00820 |
| 2490391 | 29.4901 | / | / | T<->C | / | 7/8/11/12/13/14 | non-coding region between SAOUHSC_02708 and *hlgC* |
| 1394043 | 29.1207 | SAOUHSC_01447 | *ebh* | G<->A | N<->N | 2/3/4 | extracellular matrix-binding protein |
| 615798 | 29.1128 | SAOUHSC_00625 | *mnhA2* | T<->C | I<->I | 7/8/12/13/14 | monovalent cation/H+ antiporter subunit A |
| 1842166 | 28.3065 | SAOUHSC_01933 | *hsdM* | T<->G | Y<->S | 7/12/13/14 | type I restriction-modification system subunit M |
| 361811 | 28.3065 | / | / | A<->C | / | 8/12/13/14 | non-coding region between *rpsR* and SAOUHSC_00351 |
| 399104 | 28.3065 | / | / | C<->A | / | 1/4/5/6/7 | non-coding region between SAOUHSC_00395 and 00396 |
| 1775112 | 24.8271 | SAOUHSC_01868 | *pepV* | G<->A | N<->N | 1/4/5/6/7 | dipeptidase PepV |
| 603363 | 24.7872 | SAOUHSC_00611 | *argS* | T<->A | T<->T | 1/4/5/6/7 | arginyl-tRNA synthetase |
| 448418 | 24.1427 | / | / | T<->G | / | 2/3/4 | non-coding region between SAOUHSC_00446 and 00450 |
| 1634496 | 23.9497 | SAOUHSC_01728 | — | T<->G | N<->K | 1/4/5/6/7 | hypothetical protein |
| 965494 | 22.1767 | SAOUHSC_00994 | *atl* | C<->T | K<->K | 2/3/4/5/6/7 | bifunctional autolysin |
| 84960 | 20.2605 | SAOUHSC_00079 | — | C<->T | I<->I | 2/3 | hypothetical protein |
| 2225805 | 19.5541 | SAOUHSC_02404 | — | T<->C | D<->G | 8/9 | hypothetical protein |
| 382501 | 18.8268 | SAOUHSC_00375 | *guaA* | G<->A | E<->E | 1/4 | GMP synthase |
| 2122656 | 18.4683 | / | / | C<->T | / | 8/9 | non-coding region between *ilvA* and SAOUHSC_02294 |
| 2535773 | 17.0042 | SAOUHSC_02760 | *gltB* | C<->T | T<->T | 1/4 | glutamate synthase subunit alpha |
| 271287 | 15.8725 | SAOUHSC_00251 | — | T<->A | G<->G | 1/4/5/6 | hypothetical protein |
| 590307 | 14.7232 | SAOUHSC_00591 | — | A<->T | L<->F | 4/5/6/7 | hypothetical protein |
| 2381918 | 14.2377 | SAOUHSC_02590 | — | A<->G | Y<->Y | 5/6/7/8 | hypothetical protein |
| 906471 | 14.0132 | SAOUHSC_00933 | *trpS* | G<->A | L<->L | 2/3 | tryptophanyl-tRNA synthetase |
| 1426107 | 13.9264 | / | / | C<->A | / | 5/6/7 | non-coding region between SAOUHSC_01470 and *asnS* |
| 2203557 | 13.2692 | / | / | T<->C | / | 5/6/7 | non-coding region between SAOUHSC_02381 and 02382 |
| 1019446 | 13.2402 | / | / | T<->C | / | 2/3/4 | non-coding region between SAOUHSC_01050 and 01051 |
| 368320 | 12.8523 | SAOUHSC_00360 | — | T<->A | G<->G | 2/3 | hypothetical protein |
| 2290791 | 12.8033 | SAOUHSC_02467 | *alsD* | A<->G | F<->F | 7/8 | alpha-acetolactate decarboxylase |
| 537430 | 12.6058 | / | / | G<->A | / | 3/8 | non-coding region between SAOUHSC_00532 and *hchA* |
| 584034 | 12.5499 | SAOUHSC_00581 | — | T<->C | D<->G | 2/8 | hypothetical protein |
| 1392993 | 11.8301 | SAOUHSC_01447 | *ebh* | T<->C | K<->K | 2/3 | extracellular matrix-binding protein |
| 1378107 | 11.7227 | SAOUHSC_01447 | *ebh* | C<->T | L<->L | 5/6/7 | extracellular matrix-binding protein |
| 2284683 | 11.6961 | / | / | G<->A | / | 2/3 | non-coding region between SAOUHSC_02461 and 02462 |
| 1307807 | 11.0761 | SAOUHSC_01364 | *tyrA* | G<->T | L<->M | 5/6/7 | prephenate dehydrogenase |
| 826377 | 10.8383 | SAOUHSC_00857 | — | A<->G | I<->V | 5/7 | hypothetical protein |
| 1612045 | 10.6277 | SAOUHSC_01705 | — | C<->T | A<->A | 6/7 | enterotoxin family protein |
| 2588313 | 10.6029 | SAOUHSC_02809 | *gntR* | A<->G | P<->P | 5/6 | gluconate operon transcriptional repressor |
| 1244764 | 10.5387 | SAOUHSC_01288 | — | A<->G | R<->G | 6/7 | hypothetical protein |
| 343331 | 10.4895 | / | / | T<->C | / | 5/6 | non-coding region between SAOUHSC_00330 and 00331 |
| 2451033 | 10.1731 | / | / | G<->A | / | 6/7 | non-coding region between SAOUHSC_02665 and 02666 |
| 49802 | 9.8429 | SAOUHSC_00047 | — | T<->C | F<->L | 7/8 | hypothetical protein |
| 375935 | 9.7559 | SAOUHSC_00369 | — | T<->A | E<->D | 2/3 | hypothetical protein |
| 1393088 | 9.6677 | SAOUHSC_01447 | *ebh* | A<->G | L<->L | 6/7 | extracellular matrix-binding protein |
| 1828345 | 9.591 | SAOUHSC_01919 | — | C<->T | S<->S | 6/7 | hypothetical protein |
| 2436658 | 9.3208 | / | / | G<->T | / | 2/3 | non-coding region between SAOUHSC_02649 and 02650 |
| 266885 | 8.8379 | SAOUHSC_00247 | — | A<->T | T<->S | 6/7 | hypothetical protein |
| 1401382 | 8.602 | SAOUHSC_01447 | — | A<->G | V<->A | 6/7 | extracellular matrix-binding protein |
| 1848321 | 8.567 | / | / | A<->T | / | 7/8 | non-coding region between SAOUHSC_01943 and 01944 |
| 2621187 | 8.5505 | SAOUHSC_02848 | *glcB* | C<->T | A<->A | 7/8 | PTS system glucose-specific transporter subunit IIABC |
| 1848441 | 8.5418 | SAOUHSC_01944 | — | G<->A | T<->T | 6/7 | hypothetical protein |
| 2406233 | 8.257 | SAOUHSC_02618 | — | C<->T | E<->E | 7/8 | hypothetical protein |
